# Supplementary figures and images for: An endogenously activated antiviral state restricts SARS-CoV-2 infection in differentiated primary airway epithelial cells
Source: PLoS One. 2022 Apr 18;17(4):e0266412. doi: 10.1371/journal.pone.0266412 (PMC9015133; doi:10.1371/journal.pone.0266412)

A

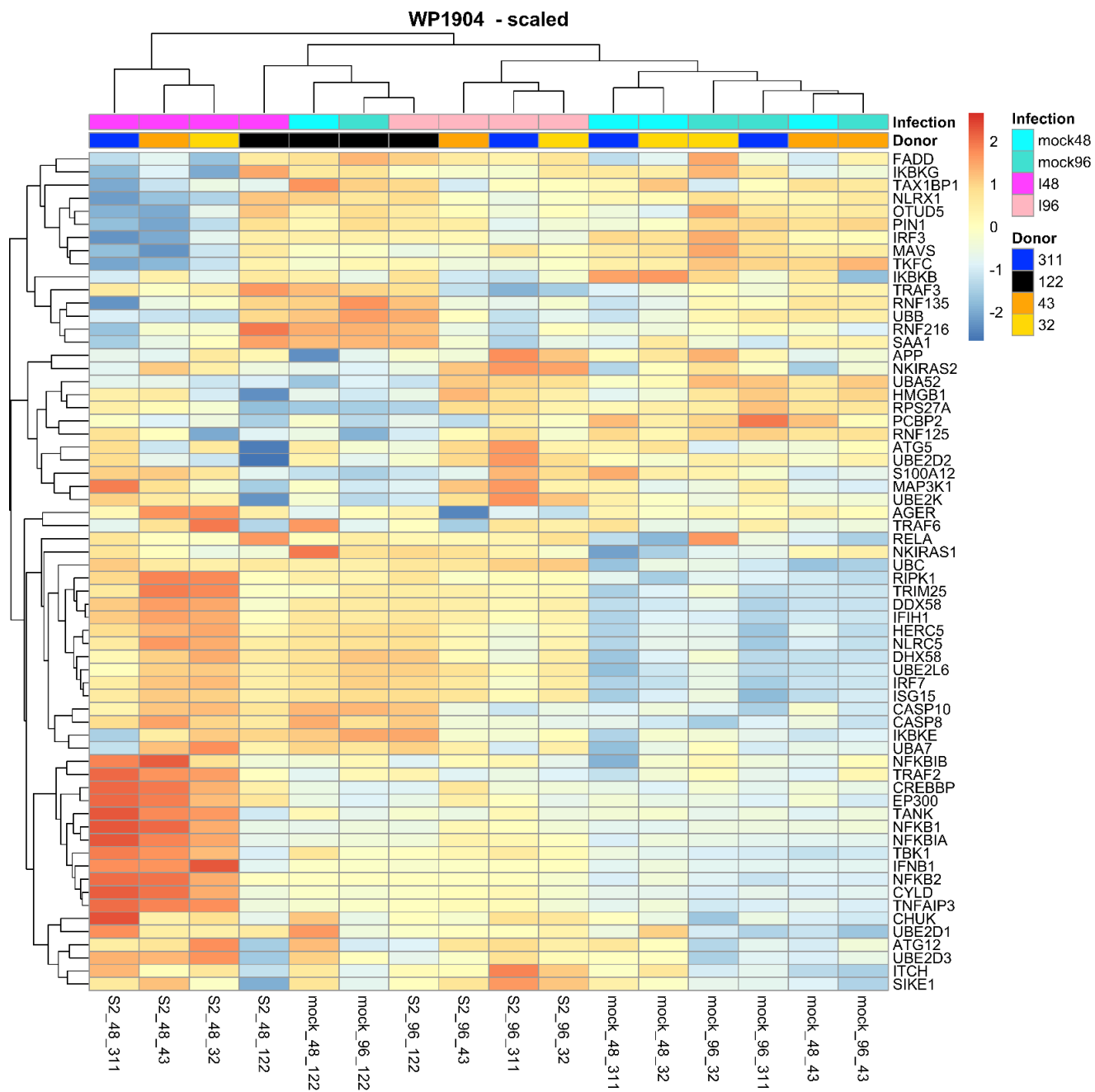

**B**

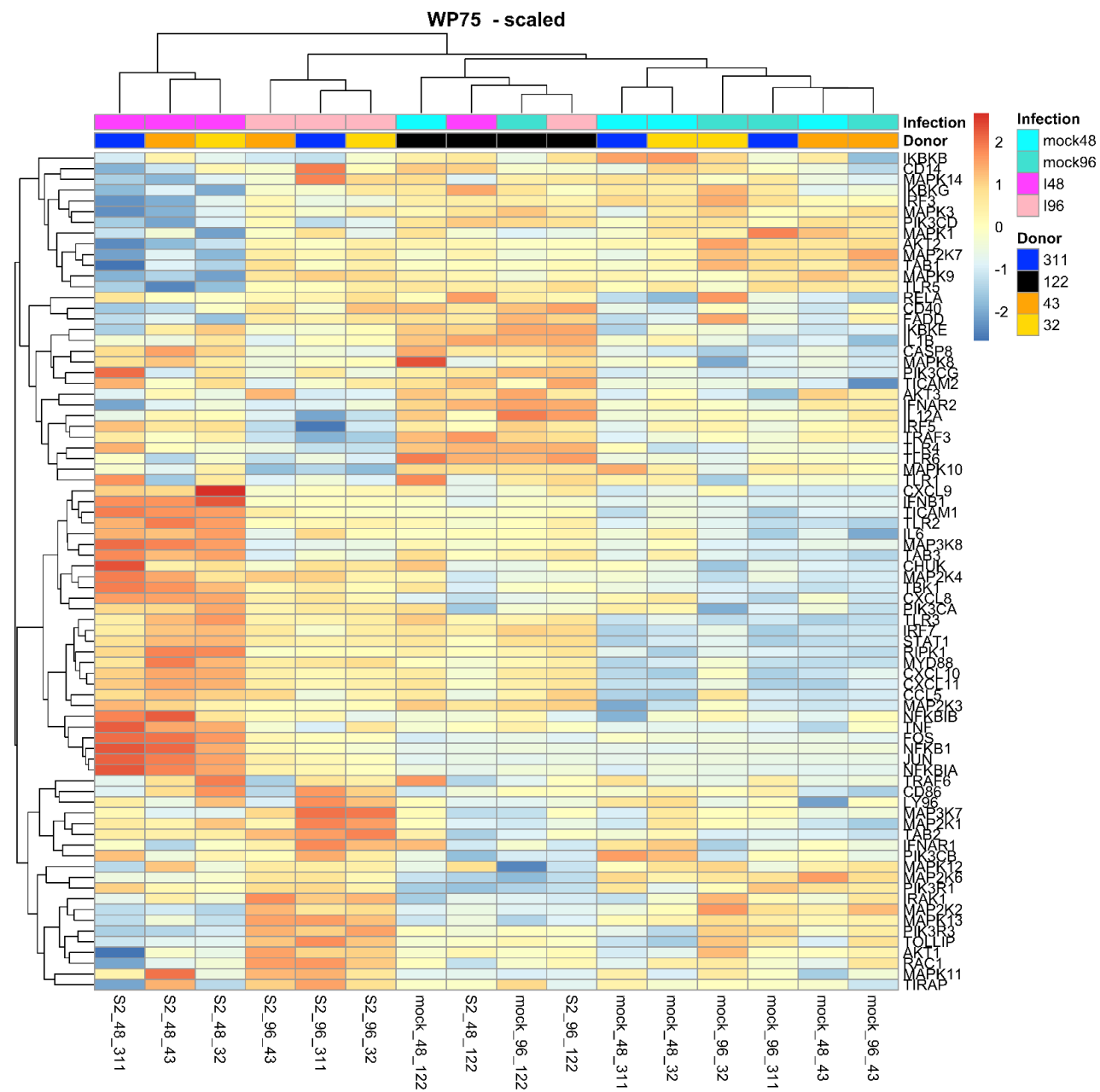

Supplement: S1 Fig — WD-PNECs from 4 adult donors were infected with a clinical isolate of SARS-CoV-2 (MOI = 0.1) or mock infected. At 48 and 96 hpi RNA was extracted from the cultures and global transcriptomic analysis was performed. Selected genes, pertaining to the IFIH1-mediated induction of interferon (A) and Toll-like receptor signalling (B) are illustrated by heatmap, gene upregulation (Red) and down regulation (blue). (PDF) [file pone.0266412.s001.pdf]
